# How to advance employment discrimination research in an era of big data and analytics

**DOI:** 10.3389/fsoc.2025.1605748

**Published:** 2025-11-10

**Authors:** Yvette P. Lopez, Helen LaVan, William M. Martin

**Affiliations:** Department of Management and Entrepreneurship, DePaul University, Chicago, IL, United States

**Keywords:** big data analytics, content analysis, empirical legal scholarship, employment discrimination research, mixed methods research, intersectionality, NVivo, qualitative research

## Abstract

This paper examines recent research on employment discrimination and addresses basic issues concerning who should be the focal subjects of employment discrimination research and which search terms should be examined. This article proposes that the way forward in employment discrimination research is using empirical legal scholarship and various large databases that support a more holistic approach to examining the different subjects of employment discrimination and the various search terms used to identify employment discrimination issues. This article explains how empirical legal scholarship, content analysis, and thematic analysis can be utilized to better understand employment discrimination. The paper concludes with propositions and recommendations for future research, including an intersectional focus.

## Introduction

One of the key controversies in employment discrimination research is the prevalence of the problem and the difficulty of researching it. Yet, research on employment discrimination has been lacking. Several studies have expressed this concern with the current state of employment discrimination research (Hajnal and Scharle, 2023; Triana et al., 2021; Derous and Pepermans, 2019; Diaz and Bergman, 2013; Ruggs et al., 2013; Joseph and Rousis, 2013; Lindsey et al., 2013).

This research focuses specifically on the United States context, where employment discrimination law operates at two levels: federal and state. At the federal level, Title VII of the Civil Rights Act of 1964, the Americans with Disabilities Act (ADA) of 1990, the Age Discrimination in Employment Act (ADEA) of 1967, and other statutes prohibit discrimination based on protected categories such as national origin, disability, gender, and race. The Equal Employment Opportunity Commission (EEOC) serves as the primary federal enforcement agency, investigating complaints and facilitating resolutions through mediation, conciliation, or litigation. When informal resolution fails, individuals may pursue claims through federal district courts, state courts, or mandatory or voluntary arbitration systems. This multi-tiered dispute resolution framework creates rich databases of discrimination cases that researchers have largely underutilized, despite their potential to provide insights into actual experiences of employment discrimination.

One of the fundamental issues of employment discrimination research is how researchers should collect the data. That is, should the focus be on employees who have experienced employment discrimination, or should the focus be on marginalized employees? We contend that archival data consists of marginalized and non-marginalized groups pursuing their rights in a given forum. Definitions vary concerning who is marginalized in a given context/situation. Are truly marginalized individuals likely to be included in other data collection methods? Does it better contribute to our knowledge of discrimination to obtain data from individuals who are actually pursuing their rights?

Identifying individuals as subjects who have been discriminated against is difficult, regardless of the basis of typically structured research (Thompson and Morris, 2013). Only a tiny proportion of individuals in any one organization have characteristics protected under the laws, and not all are necessarily discriminated against, especially within a narrow time frame (Thompson and Morris, 2013). Furthermore, it is difficult for researchers to collect the data from discriminated individuals, given top management's concern for potential legal issues and reputation. This contributes to organizations being reluctant to grant access to researchers studying the topic of discrimination (Ruggs et al., 2013). Experiencing discrimination is decidedly underreported, which further complicates the collection of the data (Cech, 2024; Bornstein, 2021; Dahl and Knepper, 2021). A recent Pew Research Poll found that nearly 80% reported seeing discrimination against Arabs, Blacks, Hispanics, as well as Jews and Muslims (Alper et al., 2024). As it relates to employment discrimination, nine out of ten (91%) of respondents said they have experienced discrimination at work, while nearly eight out of ten (77%) have witnessed employment discrimination (Monster Poll, 2023).

Given the complexities involved with gaining access to victims of discrimination in the workplace, researchers have begun emphasizing the importance of focusing on marginalized employees as a means of better understanding employment discrimination. Marginalized employees may present an opportunity for accumulating a vast body of knowledge based on their experiences with employment discrimination (Roberts and Nkomo, 2025; Ruggs et al., 2013). “Marginalization typically involves some degree of exclusion from access to power and/or resources” (Maynard and Ferdman, 2009, p. 25). Groups likely to be marginalized consist of the working poor, immigrant workers, migrant workers, young workers, chronically unemployed individuals, and “any group that has minority or lower social status in the society, including, for example, ethnic minorities, older workers, workers with disabilities, and lesbian, gay, bisexual, and transgender/transsexual (LGBT) employees” (Maynard and Ferdman, 2009, p. 26). Marginalized employees often share struggles tied to their diversity and may face challenges related to injustice, stigma, or discrimination (Maynard and Ferdman, 2009; Nikolaou, 2022).

While research has focused mainly on discrimination of marginalized groups representing ethnic, immigrant, racial, and religious minorities (Fluit et al., 2024), researchers have missed the opportunity to examine the broader range of marginalized groups' experiences (Ruggs et al., 2013) including those with disability, LGBTQIA+ and others (Fluit et al., 2024). However, some researchers have criticized the marginalized employee approach. Such criticism has focused on several dimensions, including solely focusing on marginalized groups in descriptive and mutually exclusive ways, not recognizing the complexity of discrimination from a perspective of multiple categories, and concerns of within-category discrimination (Phillips and Ranganathan, 2025; Derous and Pepermans, 2019).

Additionally, prior research examining employment discrimination has used survey investigations, which resultantly contain participants who may or may not have experienced employment discrimination. Researchers such as (Einola and Alvesson 2021) and (Wulff and Villadsen 2020) warn against overreliance on existing survey instruments when we study complex social aspects of organizations. Yet, clearly, those who have experienced discrimination in the workplace may be the richest source of information when it comes to examining employment discrimination. Therefore, it would be valuable to access the information that those who have experienced employment discrimination can provide.

A second fundamental issue of employment discrimination research involves its limited focus. As indicated above, existing research examining employment discrimination has been predominantly concentrated. It is important to examine a broader group of marginalized individuals dealing with issues related to religion, national origin, age, disability, weight, and marital status.

Yet, researchers warn about potential negative implications when several qualifying classes are examined in employment discrimination (Joseph and Rousis, 2013; Ruggs et al., 2013; Sawyer et al., 2013). That is, “casting an overly wide net in the study of discrimination may potentially make it easier for individuals to make discrimination claims based on any number of characteristics and as a result, harm social interactions in the workplaces by creating fear among employees… taken out of proportion, the study of discrimination can become muddled” (Ruggs et al., 2013, p. 256). However, this is not a justification for limiting research on marginalized groups (Ruggs et al., 2013).

In fact, we contend that there is justification in questioning whether all employment discrimination research has been adequately identified. Recently, scholars examining discrimination have begun to follow the encouragement of earlier researchers (Sawyer et al., 2013; Styhre and Eriksson-Zetterquist, 2008) on the importance of understanding intersectionality discrimination research (Vogel et al., 2018; Köllen, 2021; Mooney, 2016; Ruiz Castro and Holvino, 2016; Bailey et al., 2019). Studying multiple intersecting identities is vital to achieving a complete view of workplace diversity (Sawyer et al., 2013).

## Methodology

### The way forward in employment discrimination research

Here, we propose that researchers utilize existing databases to research individuals who have been discriminated against. This is in contrast to the use of interviews (Cuadraz and Uttal, 1999), surveys (D'Ancona, 2017), correspondence tests (Zschirnt, 2019), or experimental designs (Baert, 2018). Not that these methodological approaches fail to contribute, rather, it is more a matter of concern that they do not take a sufficiently in-depth look at examining discrimination.

This does not intend to second-guess the methodologies used by other researchers in previously peer-reviewed and published articles, but rather to note that there are certain instances where researchers could have used specific databases but did not. The contributions to discrimination research that previous researchers have made could be enhanced by using available databases, approaching the data collection and analysis through the lens of empirical legal scholarship, and using state-of-the-art analytical tools. However, the previous research is limited by these factors.

Our proposed recommendation is three-pronged: (1). Identifying societal impetus that fosters the need not only for employment discrimination research, but for research on individuals who have been discriminated against. (2). Identifying illustrative publications that have used empirical legal scholarship and these databases to research employment discrimination and/or employment-related concepts, such as whistle-blowing (Lee and Fargher, 2013), bullying (Martin and LaVan, 2010; Richardson et al., 2016a,b), or justice (Best et al., 2011), and (3). Making recommendations regarding testable premises using the recommended databases.

### Societal impetus that fosters the need for employment discrimination research with a victim focus

How researchers should conduct employment discrimination research is undoubtedly impacted by societal shifts, and legal rulings are sure to impact it. For example, concerning societal influences, the most monumental of these changes include the recent “Black Lives Matter” movement and the “Me Too” movement. It can be reasonably expected that both of these movements will result in discriminated individuals having more confidence in pursuing their rights. It can also be reasonably expected that more individuals who were retaliated against for pursuing their rights will pursue retaliation claims. Combined, these will probably lead to organizational issues of disparate impact and disparate treatment.

The contemporary political landscape further underscores the urgency of this research approach. The current administration has issued several Executive Orders (EOs) eliminating Diversity, Equity, and Inclusion (DEI) programs across federal agencies and encouraging similar changes in private sector organizations. There are three notable Executive Orders: EO 14173—Ending Illegal Discrimination and Restoring Merit-Based Opportunity (Executive Order 14173, 2025); EO 14151—Ending Radical and Wasteful Government DEI Programs and Preferencing (Executive Order 14151, 2025); and EO 14281—Restoring Equality and Meritocracy (Executive Order 14281, 2025). Despite these restrictions and changes, EEOC enforcement continues in other areas. For instance, Columbia University agreed to a $21 million settlement in January 2025 for alleged harassment against Jewish employees following October 7, 2023, the largest EEOC settlement in nearly 20 years. As a result, individual researchers and organizations may become reluctant to conduct and participate in survey-based research or qualitative research involving interviews or focus groups. Hence, archival legal databases and EEOC case records provide researchers access to discrimination experiences difficult to capture through conventional methods.

In terms of legal aspects, the Supreme Court recently ruled in RG and GR Harris Funeral Homes, Inc. V. Equal Employment Opportunity Commission (2019) that LGBTQ individuals are protected under Title VII of the Civil Rights Act of 1964. Additionally, the EEOC has recently started to recognize the validity of intersectional claims, meaning that individuals who have been discriminated against can file claims on multiple bases [Equal Employment Opportunity Commission (EEOC), 2005]. How researchers will conduct employment discrimination research in future studies will undoubtedly continue to change due to these societal and legal examples.

### Illustrations of empirical legal scholarship

Empirical legal scholarship refers to “a specific type of empirical research: a model-based approach coupled with a quantitative method” (George, 2006, p. 141). There is a subset of empirical legal scholarship that uses statistical techniques and analyses. This involves the use of studies that “employ data (including systematically coded judicial opinions) that facilitate descriptions of or inferences to a larger sample or population as well as replication by other scholars” (Heise, 2002, p. 821). Therefore, empirical legal scholarship can range from as simple as counting and surveys to more complex empirical analysis, including significance testing, multiple regression, and logit/probit analysis (Cahoy, 2010). The goals of empirical legal research are verifiability, falsifiability, and reproducibility (Bétaille, 2025).

Some authors are starting to recognize the importance and contributions of empirical legal scholarship, albeit in legal journals, and have pointed to the value of automated content analysis to enhance empirical legal scholarship (Zeiler, 2016; Allen and Blackham, 2018; Irvine et al., 2018; Blackham, 2019; Ovádek et al., 2024). We propose that the study of employment discrimination research can be enhanced by empirical legal scholarship and content analysis methodology, cognizant of both the contributions and pitfalls (Allen and Blackham, 2018).

#### Contribution of content analysis methodology

Content analysis is a methodology for discerning and organizing the content of both written and verbal forms of communication. In research related to employment discrimination, researchers have explicitly referred to the content analysis of a variety of third-party resolutions of disputes in litigation and arbitration. Initially, the content analysis of data was most likely done manually by researchers and their assistants (Evans et al., 2007; Kohlbacher, 2006). This involves manually reading and coding the cases. More recently, researchers have begun to utilize software such as NVivo and Atlas.TI, R, and/or AI (Banks et al., 2018; Jackson and Bazeley, 2019; Gibbs, 2014; Kalpokaite and Radivojevic, 2019; Miner et al., 2023; Conklin and Houston, 2025) to assist with the coding process. The efficacy of other software, such as Leximancer and Crawdad, can also be used for content analysis of this type of data (Lock and Seele, 2015). Different software require different approaches to data analysis. Most software provides online tutoring for acquisition of the skills needed to do content analysis.

Some limited research as early as the 1980s used the approach we are suggesting. Specifically, they used databases to research topics related to employment discrimination. However, they have some limitations when viewed from our current perspective, such as being dated, using relatively small sample sizes, and being narrowly focused. Examples of these few exceptions include studies examining how victims of employment discrimination fared in federal discrimination litigation related to the legal venue (Clermont and Schwab, 2004), specific litigation such as comparing case outcomes under the Americans with Disabilities Act (Posthuma et al., 2016), issues relating to contexts to discern differences in the nature of employment discrimination litigation between private and public sectors (Terpstra and Honorée, 2016) and using mixed methods in analyses of sexual harassment claims (Lockwood et al., 2011). However, these streams of research do not exist in sufficient concentration to develop theory. Additionally, it is safe to say there are thousands of employment discrimination cases arbitrated, adjudicated at the EEOC, or litigated. Yet there is a dearth of scholarly literature. One recent study found that employment discrimination plaintiffs consistently achieve worse results in arbitration than in court in the U.S., with reduced win rates, lower monetary awards, and smaller percentages of their claimed damages (Gough, 2021).

Researchers have used content analysis in their research in multiple disciplines, including psychology, sociology, law, and political science (Choo and Ferree, 2010; Dhamoon, 2011; McAllister, 2019; Pedulla, 2014; Roberts et al., 2016; Showunmi et al., 2016). Content analysis has been utilized in studies examining accounting (Grant et al., 2018); analysis of mission statements (Law and Breznik, 2018; Lopez and Martin, 2018); health care (Cronin and Bolon, 2018; Odera et al., 2016; Dilmaghani, 2022); education (Ozdem, 2011; Wilkerson and Evans, 2018); intersection of workplace violence against women and discrimination (Chuemchit et al., 2024); and corporate social responsibility (CSR) (Campopiano and De Massis, 2015; Alfa et al., 2025). Therefore, there is significant potential for content analysis in research related to employment discrimination, particularly when using litigated cases of individuals who claim to have experienced employment discrimination, which is currently lacking in the literature.

### Types of data available within the proposed databases

Researchers can use numerous existing databases to research discrimination against individuals who have reported workplace discrimination. As previously indicated, those who take action and file formal complaints of discrimination are rare (Leonard, 1984), and rulings in litigated cases can confirm whether an individual has experienced discrimination beyond claiming it. Researchers with varying levels of data analytics skills can use the databases proposed here, enabling them to research employment discrimination incidents more appropriately. Many of these data sources are readily available for little or no cost. While we describe these databases in more detail below, Table 1 provides some preliminary information about these potential data sources for employment discrimination research. Table 1 provides information describing the data sources, a description of the data contained within the data source, whether the data source is freely accessible, and whether the data source requires a subscription or other related costs. Table 1 is illustrative.

**Table 1:** Sources of data for employment discrimination research.

**Data source**	**Description of data**	**Access 1—freely available**	**Access 2—subscription**
In general		Free law project	Bloomberg, Bloomberg Law, especially Labor and Employment Practices Center
		https://free.law/2017/03/27/why-downloading-all-free-pacer/	https://pacer.uscourts.gov/
Federal litigated cases	Cases related to all aspects of federal law, not just employment, including district appellate and Supreme Court.	Google Scholar contains cases. Set settings to case law.	Bloomberg Law, especially Labor and Employment Practices Center Nexis Uni (formerly LexisNexis Academic Legal Research)
EEOC cases	Selection of EEOC cases	https://www.eeoc.gov/federal-sector/selected-noteworthy-federal-sector-appellate-decisions	Bloomberg, Bloomberg Law, especially Labor and Employment Practices Center
Arbitration	Arbitration awards by issue-criterion for inclusion unclear	Franklin County Law Library	Kluwer
		https://fclawlib.libguides.com/freewebsites/arbitration	https://www.wolterskluwer.com/en/solutions/kluwerarbitration
			American Arbitration Association
			https://www.adr.org/employment
Collective bargaining agreements	Union contracts, both private and public sectors	Department of Labor	
		https://www.dol.gov/olms/regs/compliance/cba/	
		National Council on Teacher Quality	
		https://www.nctq.org/contract-database/home	
Employer codes of conduct	Codes of expected company behaviors		Database of Business Ethics
			https://www.db-business-ethics.org/
Professional codes of conduct	Codes of expected professional behaviors Example: https://www.apa.org/science/programs/research/codes.aspx	Wikipedia–last revised in 2013	Encyclopedia of Associations
		https://en.wikipedia.org/wiki/Category:Professional_associations_by_profession	https://www.gale.com/ebooks/9781410326546/encyclopedia-of-associations-national-organizations
Individual rights cases	Particular emphasis on civil rights and free speech. Center of Individual Rights	https://www.cir-usa.org/cases/	Bloomberg, Bloomberg Law, especially Labor and Employment Practices Center
National labor relations board	Union focused-administrative law judge decisions, appellate court decisions, board decisions, and results of regional elections	National Labor Relations Board	
		https://www.nlrb.gov/	
Occupational health and safety	Litigation relating to occupational health and safety	https://www.osha.gov/safety-management/case-studies/	Bloomberg Law (includes former BNA Research Library resources) Nexis Uni (formerly LexisNexis Academic Legal Research)
Wage, hours, and leave	Litigation relating to leaves of absence	Justia	Bloomberg Law (includes former BNA Research Library resources) Nexis Uni (formerly LexisNexis Academic Legal Research)
		https://dockets.justia.com/browse/noscat-8/nos-751	
Fair labor standards act	Litigation relating to FLSA	Justia	Bloomberg Law (includes former BNA Research Library resources) Nexis Uni (formerly LexisNexis Academic Legal Research)
		https://dockets.justia.com/browse/noscat-8/nos-710	
Mental health courts	Litigation relating to individuals with mental illnesses	Substance Abuse and Mental Health Services Administration	
		https://www.samhsa.gov/gains-center/	
State courts	Litigation relating to state and municipal ordinances	Justia	
		https://dockets.justia.com/browse/state-illinois	
Freedom of information FOIA requests	Provides public access to all federal agency records – not a case database		https://www.foia.gov/how-to.html
Current population survey	Primary source of labor force statistics for the population of the United States – not a case database	https://www.census.gov/programs-surveys/cps.html	
Caveat:			
Databases are fluid and are subject to revision by the owner/administrators of the data base.			
Resources at other libraries			
http://guides.ll.georgetown.edu/home			
https://catalog.library.cornell.edu/databases/subject/,%20Resources%20Labor%20&%20Employment			

### Proposed premises for future research using the recommended databases

The databases that we propose utilizing include Federally Litigated Cases, Arbitration Cases, Collective Bargaining Agreements, and Employer Codes of Conduct.

Below, we describe examples of research involving employment discrimination that can be examined utilizing the proposed data sources. Table 2 briefly lists these data sources, associated societal impetuses, potential employment discrimination topics/concepts/terms to explore, suggested testable premises, and illustrative examples of content that may be researched from the databases. These suggestions are meant to be examples and are not intended to be comprehensive.

**Table 2:** Examples of testable premises using suggested databases.

**Data sources**	**Societal forces**	**Concepts/terms to explore**	**Suggested testable premise**	**Illustrative example of content**
Federally litigated cases	New emphasis on intersectionality and employment discrimination	Ageism, multiple motives	There are differences in outcomes in litigted cases for individuals who have more intersectional, more immutable and more observable bases for discrimination.	Katz and LaVan, 2023; LaVan and Martin, 2024
Federal litigated cases	Supreme court rulings related to LGBTQ discrimination	Workplace bullying	There are differences in outcomes in litigation for individuals who have more intersectional, more immutable, and more observable bases for discrimination.	Baumle et al., 2020; Kazyak et al., 2023
EEOC cases	Concern for employee discrimination related to health and illnesses	Various types of illness, physical and mental	There are differences in outcomes in EEOC cases for individuals who have more intersectional, more immutable and more observable bases for discrimination.	Chan et al., 2022; Leslie et al., 2020; Rumrill et al., 2022; Zilic and LaVan, 2020
EEOC cases	Retaliation related to employment discrimination	Retaliation	There are differences in outcomes in EEOC retaliation cases for individuals who have more intersectional, more immutable and more observable bases for discrimination.	Lee Badgett et al., 2018
Arbitration cases	Societal and political presence of unions influencing employment discrimination issues	Procedural justice, accommodation for illnesses, duty of fair representation.	There are differences in outcomes in voluntary arbitration when compared to mandatory arbitration for individuals who have more intersectional, more immutable, and more observable bases for discrimination.	Movahed and Hirsh, 2023
Collective bargaining agreements	Heightened public awareness for behavior of police	Constructive discharge	There are differences in outcomes of rulings based on collective bargaining agreements for individuals who have more intersectional, more immutable, and more observable bases for discrimination.	Goldhaber et al., 2016; Riccucci and Saldivar, 2014
Employer codes of conduct	Enhanced concern for integrity, transparency, and social issues related to employment discrimination	Fair treatment of employees, including fair remuneration, effective communication and learning and development opportunities	There are differences in content of Employer codes of conduct for individuals who have more intersectional, more immutable, and more observable bases for discrimination.	Kleps, 2022

### Examples of research using federally litigated cases

Federal cases are particularly useful to test a variety of hypotheses related to employment discrimination and retaliation. A few studies in the extant literature support this approach. For example, (Best et al. 2011) empirically tested litigated employment discrimination cases using a sampling frame of over 50,000 cases from 1965 to 1999, ultimately selecting a 2% random sample from both District Courts and Appellate courts. They concluded that individuals who file claims based on multiple bases do not fare as well as those filing claims based on a single protected demographic. Future research may examine how this result is likely to change given the increased societal impetus associated with intersectionality.

(Richardson et al. 2016b) researched cases in *the* federal court system to identify litigation relating to bully*ing*. They identified ninety-three cases from nine U.S. Courts of Appeal and eighty-four U.S. District Courts. Even though there is no federal law to help prevent it, protection can be provided in the court system, if individuals can prove claims such as discrimination, hostile work environment, retaliation, harassment, violations of the Americans with Disabilities Act, infliction of emotional distress, U.S. Constitution claims, disparate treatment, impact and/or discipline, wrongful discharge or termination, or violations under the Family Medical Leave Act.

### Examples of research using EEOC cases

Researchers can use EEOC cases to test hypotheses related to various types of employment discrimination. Bases for discrimination may include age, sex, race, color, religion, national origin, and disability. Recent studies that have utilized EEOC cases include (Baumle et al., 2020), who conducted a study examining 9,121 sexual orientation or gender identity discrimination charges filed with the EEOC or state or local fair employment practices agencies. They discerned trends in charge filings over time and industry patterns, issues raised, and the charges' outcomes. They specifically analyzed the differences in claim outcomes when retaliation is an issue.

(McMahon et al. 2017) conducted a study utilizing EEOC cases that looked at individuals with learning disabilities (*N* = 9,480) when compared to other general disabilities, such as physical, behavioral, or sensory disabilities (*N* = 313,480). They concluded that discrimination issues tend to involve current employees and involve harassment, intimidation, constructive discharge, and discipline. On the positive side, failure to provide reasonable accommodation and unlawful discharge are less commonplace for employees with learning disabilities.

### Examples of research using arbitration cases

Researchers can use arbitration cases to examine hypotheses related to employment discrimination and procedural justice, as well as accommodations for illnesses and/or disabilities. Past studies that have utilized arbitration cases include (Colvin and Gough, 2015), who conducted a study using the American Arbitration Association (AAA) database to discern (*N* = 10,335) case characteristics that would lead to a settlement before the arbitration hearing. In a recent study, (Gough 2021) compared employment discrimination outcomes between litigation and arbitration cases.

### Examples of research using collective bargaining agreements

Researchers can use collective bargaining agreements to examine hypotheses related to employment discrimination and issues related to transfer decisions, promotions, and constructive discharge. For example, (Riccucci and Saldivar 2014) used Westlaw to search for employment discrimination suits filed against police and fire departments due to the failure to promote between 2000 and 2011. While the authors expected to find that women and people of color are filing lawsuits against police and fire departments, they found just the opposite—most of the lawsuits filed against police and fire departments are “reverse discrimination” lawsuits filed by White men.

### Examples of research using codes of conduct

To examine hypotheses related to employment discrimination and issues related to fair treatment, researchers can use employer codes of conduct. For example, (Mazza and Furlotti 2019) analyzed the codes of ethics of companies listed on the Italian stock exchange to discern content relating to employees as stakeholders. They noted that Equal Employment Opportunity statements were more likely to be included in codes of conduct in companies with low financial distress.

As demonstrated above, there have been some studies using the proposed methodology involving various data sources, which supports how the databases listed would provide experiences of discrimination. However, that an extensive search only identified a paucity of articles that have utilized this approach in over a decade warrants mentioning. This would support our contention that these databases, yet relevant, are being underutilized. This can be potentially advantageous to increasing our understanding of employment discrimination given the increasing amount of discrimination in the workplace related to the different bases of discrimination, harassment, retaliation, mutability/immutability, communications, discipline, and intersectionality.

### Illustration of the process of content analysis

Utilizing the proposed databases above is particularly attractive given content analysis and the era of AI, big data analytics, and NLP (Waltermann and Leeuw, 2025; Brewster et al., 2014; Tene and Polonetsky, 2013). To illustrate how researchers can implement the analysis using the suggested databases, consider examining disparate impact in the workplace. Disparate impact may exist when a seemingly neutral policy or practice disproportionately negatively impacts protected individuals. This raises the question of whether there are protections for the bases under the law.

To discern how disparate impact has been conceptualized and researched in the literature, researchers should consult prior research on disparate impact. It is not likely that prior research will have used the recommended databases and empirical legal scholarship. This underutilization of existing databases is one of the main points of this manuscript. Researchers could test this type of hypothesis using federally litigated or arbitrated cases. The researchers should be looking for terms that will be in the case content. At this point, researchers should confer with subject matter experts to help formulate the concepts. In addition, some content analysis software enables the use of trend analysis to discern trends in the data that might not be obvious to human experts. Content analysis software provides output in the form of frequencies of terms.

Researchers can use content analysis software to query the cases for the relevant terms. In the content analysis process, it is possible to identify synonyms for the relevant terms that are coded. It is also possible to eliminate terms that do not have their intended meaning in the given context, such as the term “Black” which could be the name of an individual or a company. Then the researcher can identify the variation in the cases to test the premise. It is possible to use a graphic portrayal of the data and multivariate nonparametric statistics to analyze the data. The results of this research could be structured in such a way as to provide changes in human resource management and organizational change processes.

## Discussion

Most previous publications have not used individuals who have been discriminated against as the unit of analysis, regardless of the strategy used. This is even though there are multiple sources of readily available data. There have been significant advances in methodologies, including software, that facilitates handling big data sets with more sophisticated statistical methodologies, including principal component analysis and cluster analysis. Our discipline should be more accepting of the viability and contributions of empirical legal scholarship, and the use of big data and related analytics.

In the U.S. context, given the political and legal headwinds regarding DEI and employment discrimination at both federal and organizational levels, our proposed approach offers researchers a pathway to continue advancing our understanding of employment discrimination. These databases capture the experiences of individuals and collective aggregate populations who have navigated the formal legal system, providing insights that remain accessible despite these contextual changes.

The proposed research approach of using empirical legal scholarship and content analysis to enhance employment discrimination research not only contributes to practice, but also to theory. Specifically, researchers can apply a grounded theory methodology given the proposed qualitative research methods approach (Morse, 2009). This enables the discovery of inductive theory (Wiesche et al., 2017). Grounded theory “allows the researcher to develop a theoretical account of the general features of a topic while simultaneously grounding the account in empirical observations or data” (Martin and Turner, 1986, p. 141).

A major emphasis of this article is that employment discrimination research should be conducted on employees who have experienced discrimination. Researchers might expect some pushback due to inconsistencies in reporting. However, no method is without some limitations. This is not significantly different from data gathering issues such as interviewer bias, non-respondent bias, and coding errors. Moreover, experimental designs may contain contaminating variables that are not controlled. Scholars recognize that using litigants in the sample represents the extreme end, when other internal dispute resolution mechanisms have failed. However, given the large sample sizes that are possible with these databases, knowledgeable court staff can review case write-ups to obtain accurate data. Also, the auto-coding and the analysis of trends in NVivo (for example). Auto-coding and trend analysis in NVivo (for example) increase insight into the data and reduce human coding errors.

Data analytics is offering researchers of employment discrimination the opportunity to research the complexity of discrimination in a systematic, scientific, and comprehensive manner. Based on an examination of the literature, experimental design and surveys are two methodologies that have been utilized extensively by management researchers.

Multiple case studies, analyzing one or a few cases at a time, have been used significantly by legal researchers. Yet, big data analytics allows for both managerial and legal issues to be examined more comprehensively based on real instances of discrimination.

### Limitations

The proposed methodology faces challenges, including the fact that some cases remain unpublished, making it unclear how well the published articles are representative. It has been contended that there are other methodological issues, such as the fact that the write-up of a case is *post hoc*, there are changes in the law over time, and researchers use inappropriate statistics and data aggregation methods. Additionally, safeguards are needed to protect subjects, especially when databases are shared.

### Research context

The U.S.-specific nature of this research presents both strengths and limitations. While the federal legal framework and multi-tiered dispute resolution system create comprehensive databases, the findings may have limited generalizability to other national contexts, cultural norms, or discrimination complaint processes. Additionally, the current political climate's impact on DEI programs and discrimination reporting may influence the types and frequencies of cases appearing in these databases, potentially creating temporal bias that researchers must consider when interpreting trends and outcomes.

### Published vs. unpublished cases

Published vs. unpublished cases as a concern is not dissimilar from non-respondents in a survey. In fact, this is not a new issue (Siegelman and Donohue, 1990; Swenson, 2004). This concern is more or less accepted as a limitation. Some publications have offered insight into unpublished cases (McAllister, 2019) and some researchers have been able to combine both published and unpublished cases in their analysis (Cooper and Barrett, 1984). There is even a contention that there is discrimination in how decisions are made regarding which cases get published and which cases do not get published (Tillman and Hinkle, 2018).

### Issues with policy capturing

Ultimately, the methodology involved in this proposal is a form of policy capturing, which has been linked with contributions and limitations regardless of the subject matter. Early research has cautioned using cases for policy capturing (Roehling, 1993). In some regards, this caution can be briefly summarized as follows: The write-ups of the cases are *post hoc*, the evolving nature of the laws present sampling problems, the statistics involved may be too simple at times and too complex at other times, there can be systematic sampling biases, and there may be incorrect aggregation of the data.

Yet, we believe that these cautions are controllable in the data collection and analysis process. First and foremost, one has to take the position that while not all cases are published, the ones that are published have a greater impact on future litigation and policy-making. Additionally, the cases are frequently reviewed by more than one individual before publication. They frequently have expert witnesses, friends of the courts, and other documents to supplement the judges' rulings. One way of overcoming changes in the law over time is to have a shorter sampling time frame, and ideally, a more recent time frame. Additionally, the more widespread use of nonparametric statistics can overcome some of the statistical issues. Moreover, since (Roehling 1993), authors have contended (and reviewers and editors have agreed) that these are not insurmountable challenges (Clermont and Schwab, 2009; Goldhaber et al., 2016; Johnson et al., 2008; Knapp and Heshizer, 2001; McMullen, 2016; Zschirnt, 2019; Hajnal and Scharle, 2023).

### Methodological progress

Methodologists have been working to develop techniques to improve research using policy capturing (Aiman-Smith et al., 2002; Karren and Barringer, 2002) and large databases. As with other methodologies, methodological issues are consistently being addressed by methodologists, such as: the handling of missing data (Bonaccio and Dalal, 2010); the issue of socially desirable responses in self-reporting (Tomassetti et al., 2016), the issue of multi-collinearity (Paetzold, 1992), and the ethics in the use of correspondence studies (Zschirnt, 2019). Methodologists have also worked on issues relating to large databases (Ross et al., 2018). These authors have pointed out the challenge of sharing of data sets and still maintaining the privacy of the research participants (Ross et al., 2018).

It should be noted that some of these concerns are not applicable to the methodological approaches being discussed in this manuscript. There are absolutely no concerns regarding social desirability, the ethics of correspondence studies (Zschirnt, 2019) and the protection of subjects. Moreover, the data in these databases are considered archival, and an internal review by an Internal Review Board (IRB) is typically not required. In some of the databases, however, some identifying data is redacted.

### Future recommendations

First and foremost, our recommendation would be to use the large databases that are readily available, some of which are free, to research employment discrimination. We have identified at least 25 sources of this data type, but surely there are more. Additionally, we have suggested approaches to developing testable hypotheses utilizing the recommended data sources.

Future research should examine how current political and organizational headwinds on DEI programs and labor immigration policies influence discrimination patterns, complaint-filing behaviors, and case outcomes with these databases. Additionally, comparative studies examining discrimination patterns before, during, and after major events could provide valuable insights into how policy environments shape employment discrimination experiences.

Researchers in our discipline should learn from researchers' experiences in other disciplines, especially psychology, sociology, political science, gender studies, and law. It should be noted that some of these publications are in the top journals of their respective disciplines or subdisciplines.

## Conclusion

The problem of improving research in employment discrimination has been addressed for at least the past 15 years. It is a multinational issue. Although the focus of this research has primarily been on data collection in databases in the U.S., (Foster and Williams 2011) noted the existence of intersectionality and its legal aspects in employment discrimination research in Great Britain. Moreover, the suggestions, perhaps even the admonishments, have considered broadening the categories on which the discrimination could be based, i.e., intersectional bases, and including contextual factors.

Cortina and Kirkland's (2018) perspective is that intersectionality and what they entitle double jeopardy (meaning employment discrimination disputes filed on more than one basis), persist in complicating discrimination research. However, this double jeopardy reflects reality (Sicenica, 2023). They suggest new approaches to conducting the research. These include newer, interdisciplinary fields that offer boundary-spanning vantage points, promising to move discrimination research in new directions. These include such fields as…sociolegal studies…and disability studies.

(Outtz 2018) considered whether scholarly work in discrimination can make a practical difference. He noted that much existing research on discrimination does not give sufficient consideration to the factors that underlie discrimination or defines the target group too narrowly.

(Triana et al. 2021) recommended expanding the focus of discrimination research to other target categories (i.e., outside of sex and race) that have received comparably less attention. Additionally, they suggest broadening the scope of discrimination research beyond commonly studied contexts (e.g., recruiting, selection, and pay) to examine where, when, and how discrimination occurs more covertly.

(Stainback 2018) sums it up perfectly: “Our ability to move beyond a description of what happens to a focus on identifying the factors that might be most effective in reducing status-linked inequalities would renew the relevance of academic scholarship for real-world workplaces” (p. 53).

In conclusion, we believe that research on employment discrimination can be enhanced by utilizing empirical legal scholarship and content analysis software to analyze large, publicly available databases. Recent enhanced societal awareness of the subjects of discrimination and harassment, as evidenced by reported incidents and multimillion- dollar lawsuits, would allow the proposed methodologies to make significant contributions to the understanding, theory, practice, and potential avoidance of employment discrimination.
